# Supplementary material for: Understanding the microbial biogeography of ancient human dentitions to guide study design and interpretation
Source: FEMS Microbes. 2022 Mar 3;3:xtac006. doi: 10.1093/femsmc/xtac006 (PMC10117714; doi:10.1093/femsmc/xtac006)
Supplement: xtac006_Supplemental_Files [file xtac006_supplemental_files.zip › supplemetary_figure_legends.docx]

**Figure S1.** Photos of the entire available dentitions of the four individuals sampled in this study.

**Figure S2.** Sourcetracker results, generated from a genus-level OTU-table.

**Figure S3.** Inverse Simpson Index by mass of the original calculus deposit.

**Figure S4.** Damage of first base at the 5’ end of fragments mapping to *Tannerella forsythia*.

**Dataset S1.** Study metadata and OUT tables
