# Supplementary figures and images for: Understanding the microbial biogeography of ancient human dentitions to guide study design and interpretation
Source: FEMS Microbes. 2022 Mar 3;3:xtac006. doi: 10.1093/femsmc/xtac006 (PMC10117714; doi:10.1093/femsmc/xtac006)

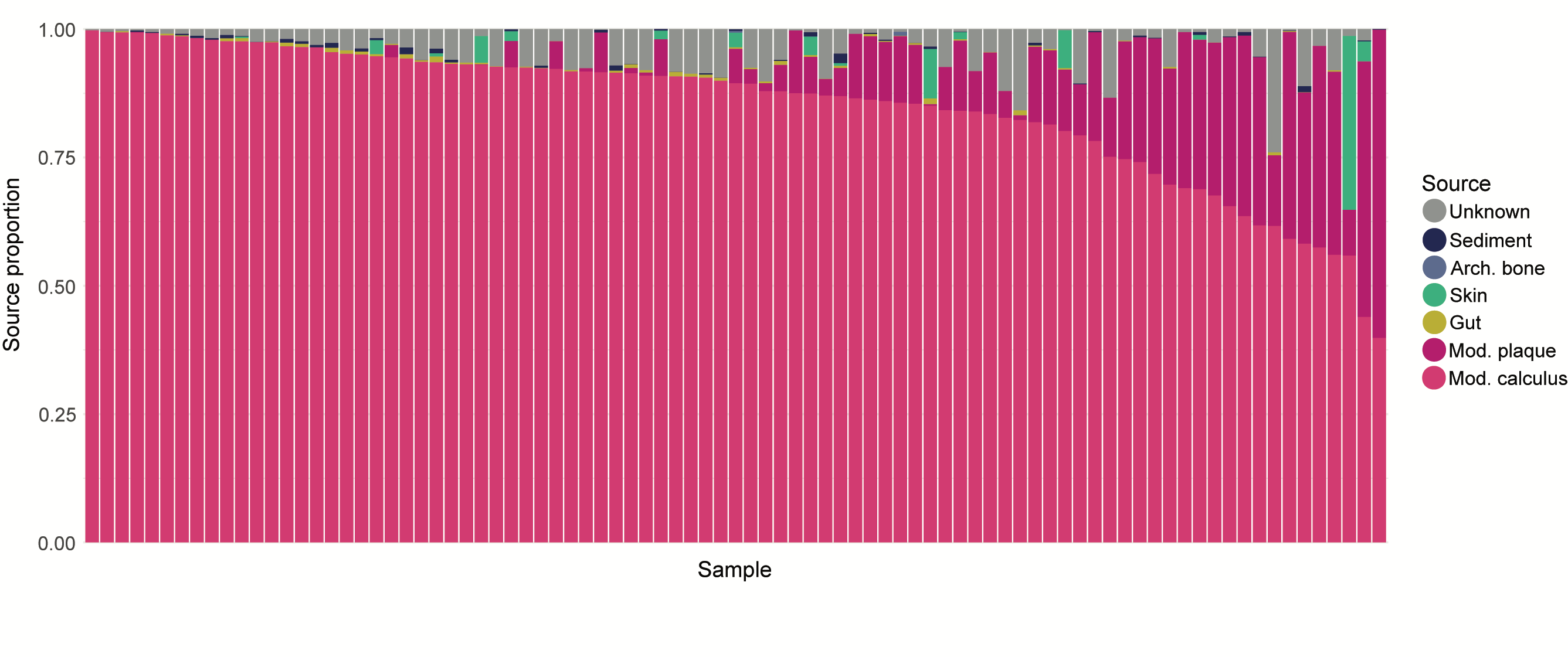

Supplement: xtac006_Supplemental_Files [file xtac006_supplemental_files.zip › S2_sourcetracker_shotgun_genus_20210617-5.tiff]

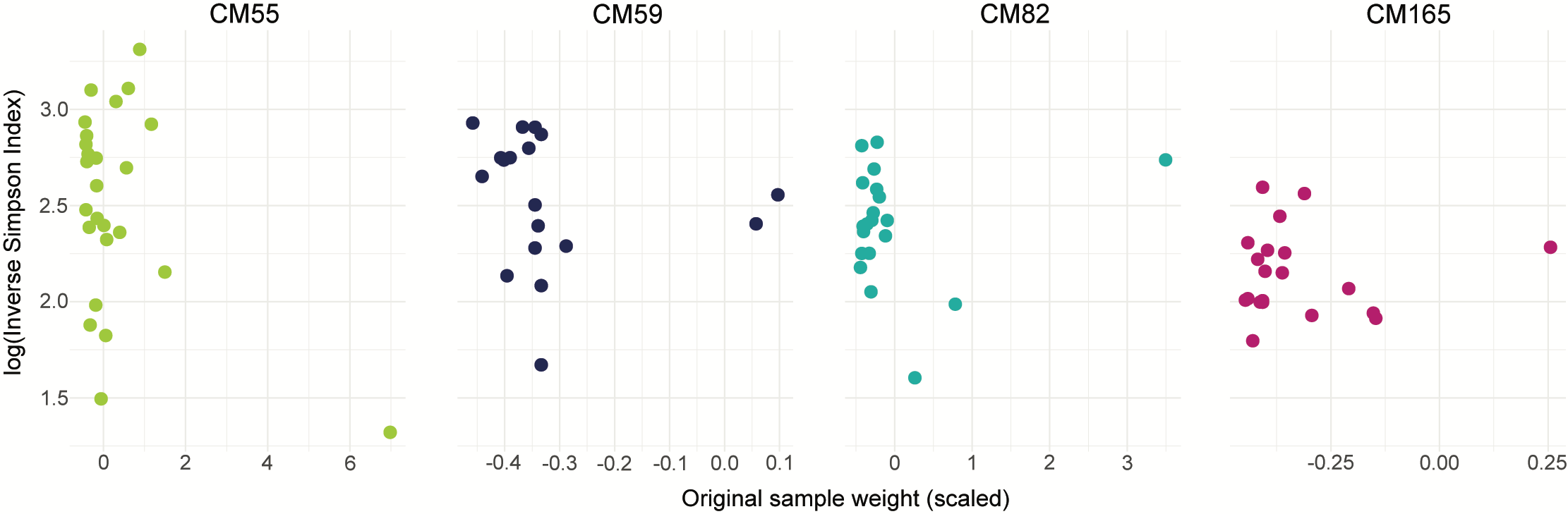

Supplement: xtac006_Supplemental_Files [file xtac006_supplemental_files.zip › S3_simpson_weight_20210408-5.tiff]

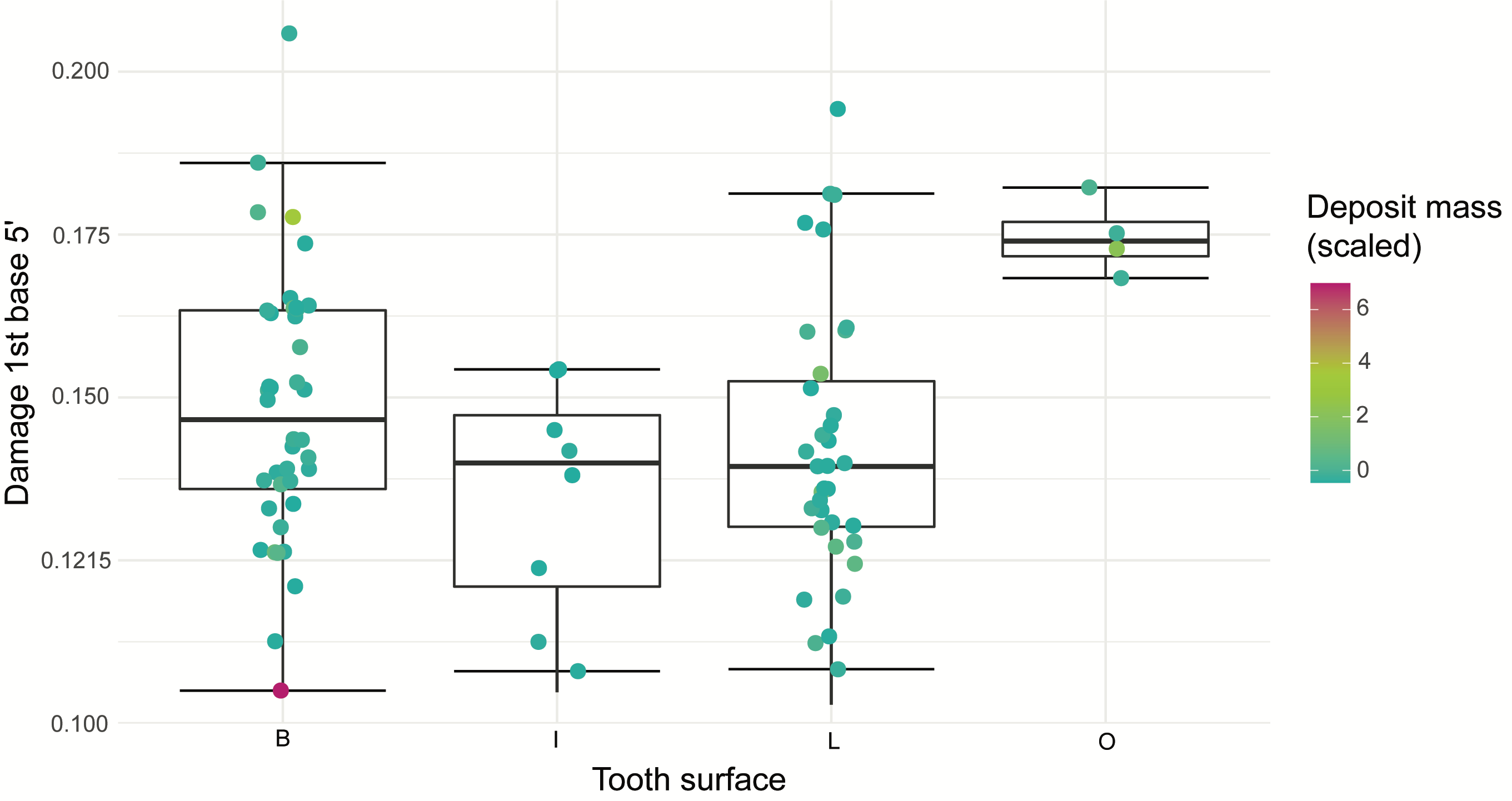

Supplement: xtac006_Supplemental_Files [file xtac006_supplemental_files.zip › S4_damage_fig_20210609-5.tiff]

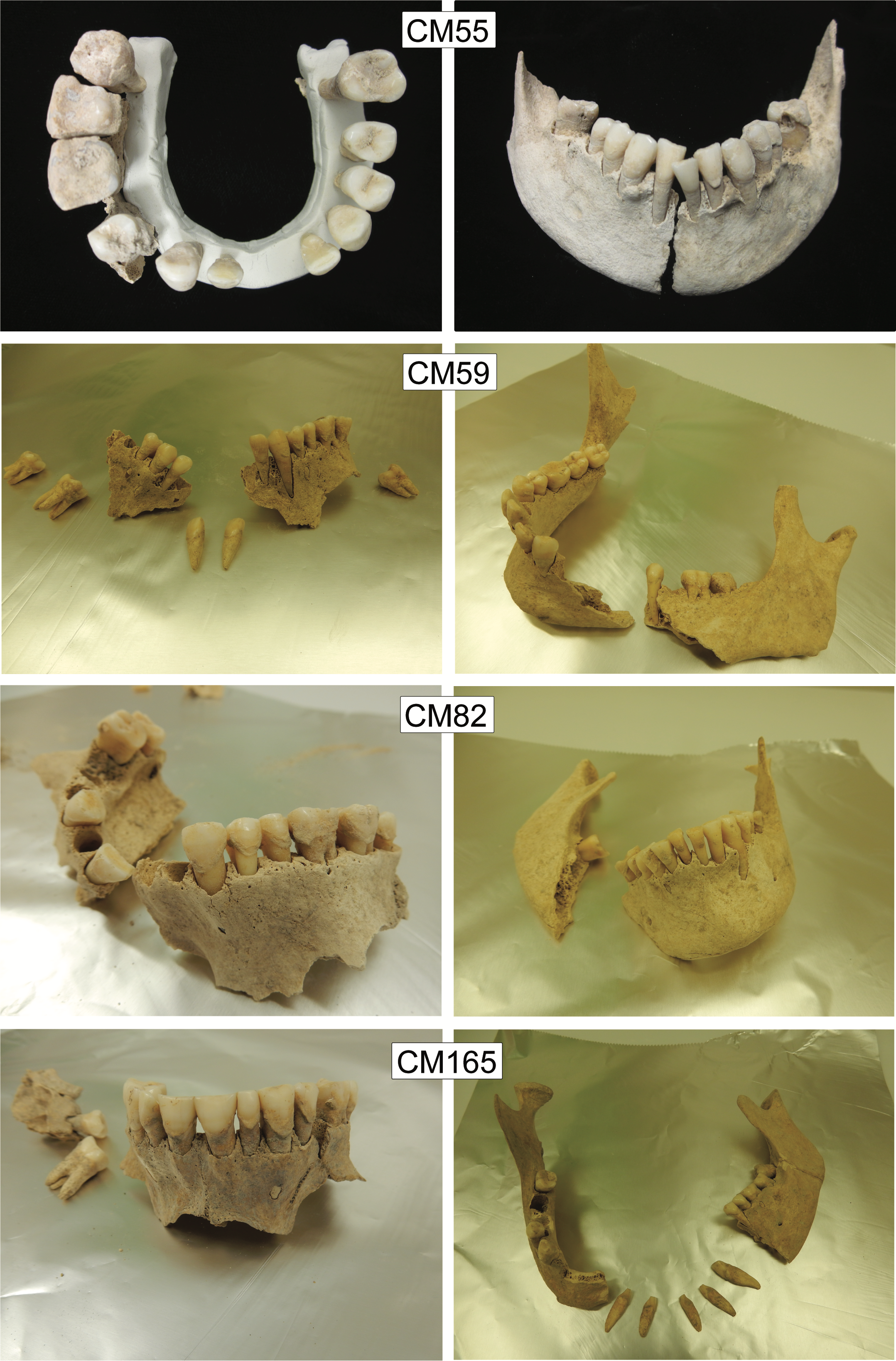

Supplement: xtac006_Supplemental_Files [file xtac006_supplemental_files.zip › S1_dentition_photos-5.tiff]
