# Supplementary material for: Understanding the microbial biogeography of ancient human dentitions to guide study design and interpretation
Source: FEMS Microbes. 2022 Mar 3;3:xtac006. doi: 10.1093/femsmc/xtac006 (PMC10117714; doi:10.1093/femsmc/xtac006)
Supplement: xtac006_Supplemental_Files [file xtac006_supplemental_files.zip › FEMSMC-2021-045.R1_R2R.docx]

Point-by-Point Response to Reviewer Comments for FEMSMC-2021-045
“Understanding the microbial biogeography of ancient human dentitions to guide study design and interpretation”

We received six specific comments from two reviewers. We present our responses to each reviewer comment below. Changes to the text have been marked in blue in the manuscript file.

Reviewer #1 **Hannes** **Schroeder** University of Copenhagen (Remarks to the Author):

Comment 1: This is an excellent manuscript which makes an important contribution to the field of ancient oral microbiome studies. It is well known that ancient dental calculus provides an excellent reservoir of ancient microbial DNA that can be used to study the evolution of the human oral microbiome. Most archaeological studies of ancient dental calculus rely on a single sample per individual taken from the oral cavity and it is generally assumed that this sample is a good representation of the entire oral cavity. However, it is unclear if this sampling strategy introduces biases and that is what this study seeks to address. Fagernäs et al. used shotgun sequencing to analyse 87 archaeological dental calculus samples from different locations in the oral cavities of four individuals with low to heavy dental calculus deposits from the Iberian Chalcolithic site of Camino del Molino (ca. 4500-5000 BP) in Spain. Samples were taken across the entire dental arcade, including different tooth types (incisor, canine, premolar, molar) and tooth surfaces (buccal, labial, interproximal, occlusal). Following initial characterisation of the ancient oral microbiomes using established pipelines (MALT, Sourcetracker etc), the data were analysed using principal component analysis and multivariate analysis of variance (PERMANOVA) to explore differences in composition and function between individuals, tooth surface, tooth position, and deposit mass. Results indicate that the main source of variation in oral microbiome composition and function is the sampled individual, although minor differences in community composition and functional potential could also be detected with respect to tooth position, deposit size, and tooth surface. The authors conclude that one randomly selected sample can, for most purposes, be used to represent an individual in population-level comparative studies.

The sampling strategy, experimental setup, and analytical methods appear to be entirely appropriate to the question and the results (and therefore the authors conclusions) seem to be solid and I have very little to add in terms of critique. So, what follows are merely some suggestions and questions directed at the authors:

Response: We thank the reviewer for this positive assessment of our manuscript.

Comment 2: Your main finding is that the main source of variation in the composition and function of the ancient oral microbiomes you recovered is the sampled individual and the formal statistics back that up. However, looking at the PCAs I can’t help thinking that there is still quite a large amount of variation within individuals as well (as indicated by the low R2 values). So, in other words if we took a random sample from each of these four individuals we might end up with quite different compositional and functional (?) profiles which might be misleading. So, are we perhaps underestimating within-individual differences here? I am not suggesting that you should revise your conclusions, but I am having trouble reconciling the PCAs and the formal statistics and I wonder if it’s worth commenting on that in the text.

Response: We have edited the text to reflect this important point. On line 359 we have added “We observe variation in microbial community composition both within and between individuals”, and on line 360 we have added “however, the R2 value is very low, indicating that other unknown factors contribute to this variation”.

Comment 3: In Figure 5, you chose not to display a PCA of the functional profiles plotted by tooth position, as you did in Figure 4 - why not? Also, I was wondering what the PCAs would look like if you tried to combine them, i.e. showing individuals (by color) and tooth position (by shape) in one plot. I know it would make the plots quite busy, but I was wondering if you would see other patterns emerging.

Response: We have added a new figure panel (5B) showing the factors significantly affecting functional potential. In general, combining the PCAs makes it more difficult to see the the minor patterns, and no new patterns emerge, which is why we chose not to combine the PCAs showing individuals with any of the other significant factors.

Comment 4: You also find a positive correlation between deposit mass and DNA damage, which is an interesting finding in itself, but (as far as I could tell) you do not discuss this finding any further in the text. But I would be interested to hear your thoughts, so would you be willing to offer a possible explanation? For example, do you think it is simply due to the fact that larger deposits include more ancient (authentic) DNA or are there maybe other mechanisms at play in connection with the slow build-up of dental calculus deposits in the oral cavity?

Response: We have added a section in the discussion relating to this pattern, starting on line 535: “In addition to occlusal samples having an increased amount of DNA damage, an association was also found between deposit mass and DNA damage for other sampling sites. This pattern may be related to a greater immune response at sites with large dental calculus deposits, or possibly be due to the lower density that is common for large deposits, but more research is necessary in order to understand this association.”

Comment 5: The functional profiling is interesting and backs up your conclusions, but I was wondering if the results would change if you consider complete pathways (I am assuming here that you are not due to low coverage).

Response: Unfortunately, in our experience, studying complete pathways would require both very deep sequencing depths, as well as only using longer fragments from the dataset. We therefore cannot adequately address this question with our current ancient dataset.

Comment 6: Your conclusion is rather short and some of the additional findings (e.g. regarding diet, damage patterns etc) are not mentioned again which is a bit of a shame as they are all interesting findings. So, I suggest expanding the conclusion to include some of the other findings. If the text becomes too repetitive, I suggest moving some of the “conclusions” from the last part of the introduction to the conclusion.

Response: We have expanded the conclusion paragraph, as well as moved parts of the last paragraph of the introduction to the conclusion, as suggested.

Reviewer #2 Egija  **Zaura** ACTA (Remarks to the Author):

Comment 1: This is a very well written manuscript on the effects of intraoral location and sample mass on ancient human calculus composition and microbial functional potential. The authors conclude that the differences within an individual are small in comparison to interindividual differences and assessment of a calculus sample from a single intraoral location is appropriate for future studies.

Response: We thank the reviewer for their positive assessment.

Comment 2: One issue is not sufficiently highlighted and remains unclear to me: what about the difference between the supra- and subgingival calculus? The authors focus on different teeth or buccal vs lingual or interproximal sites, while ecologically largest differences in dental plaque are observed between the part that is above the gum line (supragingival) and that below – subgingival. I expect the same for the dental calculus. Dental calculus (during one’s life) even has a different color depending on the source of plaque liquid: subgingival is dark, greenish due to presence of heme and is composed mainly of anaerobes, while supragingival is white-yellow, and mainly composed of facultative anaerobes and aerobe taxa. The reason for calcification is also different: supragingival calculus is formed near salivary duct orifices such as lower lingual incisors and upper buccal molars, while subgingival calculus is more generalized and would form where gingival sulcus is large enough for undisturbed accumulation of plaque, such as in untreated periodontitis cases. The results from the occlusal calculus in the current study suggest that this might have been supragingival calculus, near the orifice of parotis gland. The methods (p. 4 line 45) describe sample collection as: “….supragingival dental calculus was separately collected from four different surfaces on each tooth…”. It should be described in more detail for the reader to be able to follow how the distinction between the supra- and subgingival calculus was done, knowing that there was no gingiva present. How could authors be sure that it was supragingival calculus they collected? Even if the cemento-enamel junction (CEJ) was used to discriminate this, one cannot assume that gums started below this junction. In cases with alveolar bone destruction as in some of the cases here, one cannot use CEJ for this due to gum recession during life.

Response: We have added a clarification to the methods as to how the sampling was performed starting on line 165, “During sampling, we collected dental calculus deposits from enamel surfaces, above the apparent gingival margin, thereby limiting sampling to supragingival calculus. In some cases, gingival recession caused supragingival calculus to extend slightly beyond the cementum-enamel junction and small quantities of subgingival calculus may have been unintentionally sampled together with the overlying supragingival calculus. However, we attempted to avoid such cases, and incidental inclusion subgingival calculus was likely very minor, especially due to the generally good dental health of the individuals.”

In general, the described coloration differences between supra- and subgingival dental calculus do not persist in the archaeological record (the pigment compounds break down), and can therefore not be used to distinguish calculus types. However, it is often possible to determine where the gingival margin was at the time of death based on the shape and position of the dental calculus deposit, as can be seen on several of the teeth in Supplementary Figure 1. We attempted to sample only calculus attached to enamel, but supragingival calculus can begin to overgrow subgingival calculus as the gingival margin recedes. We attempted to avoid such cases, and believe that any incidental inclusion subgingival calculus was very minor, especially due to the generally good dental health of the individuals, as described in detail in the Material and Methods section.
